# Bad Breath

**Published:** 1875-06

**Authors:** 


### BAD BREATH.

Nothing is more offensive to those about us than a bad breath, and nothing is more easily remedied. To be sure there are many cases in which the remedy must be almost constantly employed; but these are rare as compared with the aggregate, and demand somewhat radical measures. Nine persons out of ten can keep their breath sweet and pure and their mouths free from offensive exhalations, by thoroughly using Dr. Lyon's Tooth Tablets, each morning and night, with the stiffest of stiff brushes. Especially should these be used the last thing at night, and, after being used, the mouth should not be rinsed, as the particles of this dentifrice remaining about and between the teeth, serve to neutralize any acid which may be found from the decomposition of such specks of food as the brush has failed to remove.

We say this treatment would sweeten the breath and preserve, in beauty and usefulness, nine sets of teeth in ten. We say further, that there is no mouth, however offensive, which may not be vastly improved in condition, by the regular use of this article, morning and evening. There are thousands of cases of bleeding gums, and loosened teeth, which may be cured by this very simple treatment. But when the bad odor arises from great accumulations of salivary calculus or "tartar," which always induces ulceration of the gums when allowed to accumulate largely and to insinuate itself under its free edge—or from a dead tooth from which pus constantly exudes, there must be such radical treatment as only a skillful dentist can supply, before absolute sweetness and purity are secured. Still, in all cases, however offensive, the Tablets will greatly lessen the terrible evil. We call it a "terrrible evil," and we will endeavor to allude to some of its terrors hereafter.

---

### "MISFIT CARPETS."

Thousands have noticed the above line at the head of a brief advertisement by Mr. M. S. Bendall, of No. 112 Fulton Street, New York. It is curious to notice what a large business this gentleman has built up, in a business never before prosecuted. His warerooms are the repositories of all the carpets which are cut off and made up, and not paid for by the purchasers, in New York, Philadelphia, and Boston. Somebody in Ohio orders from a leading dealer a fine carpet, to be cut and made and sent C. O. D. When it reaches him he can not pay the bill. Of course the express takes it back. It has been cut for a peculiar-shaped room, and is no longer a salable piece of property. It finds its way next day to Bendall; and at his store, to which people flock for bargains, it is seen and bought by some one whose room it just happens to fit, at 25 or 30 per cent under price. This is a great convenience to the world, and economical buyers avail themselves of it. Mr. Bendall is a high-toned gentleman, cultivated and refined, and his success has been—as it ought to be—great.

---

The article intended as a sequel to the one entitled "Self-Inflicted," in our May number, and which will bear the title, "Self-Remedied," will appear in the *Journal* for July.

